# A single introduction of *Yersinia pestis* to Brazil during the 3^rd^ plague pandemic

**DOI:** 10.1371/journal.pone.0209478

**Published:** 2019-01-09

**Authors:** Amy J. Vogler, Jason W. Sahl, Nilma C. Leal, Marise Sobreira, Charles H. D. Williamson, Molly C. Bollig, Dawn N. Birdsell, Andrew Rivera, Brian Thompson, Roxanne Nottingham, Antonio M. Rezende, Paul Keim, Alzira M. P. Almeida, David M. Wagner

**Affiliations:** 1 The Pathogen and Microbiome Institute, Northern Arizona University, Flagstaff, Arizona, United States of America; 2 Institute Aggeu Magalhães, Recife, Pernambuco, Brazil; 3 Translational Genomics Research Institute North, Flagstaff, Arizona, United States of America; University of Florence, ITALY

## Abstract

*Yersinia pestis* was introduced to Brazil during the third plague pandemic and currently exists in several recognized foci. There is currently limited available phylogeographic data regarding *Y*. *pestis* in Brazil. We generated whole genome sequences for 411 *Y*. *pestis* strains from six Brazilian foci to investigate the phylogeography of *Y*. *pestis* in Brazil; these strains were isolated from 1966 to 1997. All 411 strains were assigned to a single monophyletic clade within the 1.ORI population, indicating a single *Y*. *pestis* introduction was responsible for the successful establishment of endemic foci in Brazil. There was a moderate level of genomic diversity but little population structure among the 411 Brazilian *Y*. *pestis* strains, consistent with a radial expansion wherein *Y*. *pestis* spread rapidly from the coast to the interior of Brazil and became ecologically established. Overall, there were no strong spatial or temporal patterns among the Brazilian strains. However, strains from the same focus tended to be more closely related and strains isolated from foci closer to the coast tended to fall in more basal positions in the whole genome phylogeny than strains from more interior foci. Overall, the patterns observed in Brazil are similar to other locations affected during the 3rd plague pandemic such as in North America and Madagascar.

## Introduction

*Yersinia pestis*, etiologic agent of plague, is one of the most successful zoonotic pathogens known. Molecular investigations have revealed that *Y*. *pestis* spread multiple times from its original foci in Central Asia [1–3] to cause three recognized pandemics: Justinian’s plague in the 6^th^– 7^th^ centuries, medieval plague in the 14^th^– 17^th^ centuries (including the Black Death), and the third pandemic, which began ~1855 in the Chinese province of Yünnan [4]. Each of these pandemics was marked by increasingly successful spatial dissemination of *Y*. *pestis*, eventually leading to the worldwide distribution of natural foci observed today [3, 4]. Although typically well controlled via improved hygiene and the widespread availability of antibiotics and insecticides, *Y*. *pestis* remains a human health threat due to the severity of the disease, the many established natural plague foci [4], and its potential for use as a bioterror agent [5].

*Y*. *pestis* was first introduced into Brazil during the third pandemic, when much of the current worldwide distribution of *Y*. *pestis* was established [4]. However, the exact entry point and source of the foci that became established in Brazil are not known. The most commonly reported introduction point is the port of Santos, São Paulo (SP), where there were confirmed human plague cases following reports of a murine epizootic in 1899 [6]. Other Brazilian ports also experienced plague outbreaks over the next seven years, including ports in Ceará (CE) and Rio de Janeiro (RJ) in 1900; Pernambuco (PE) and Rio Grande do Sul (RS) in 1902; Pará (PA) in 1903; Bahia (BA) in 1904; and Espírito Santo (ES), Paraná (PR), and Sergipe (SE) in 1906. The ports of Paraíba (PB) and Alagoas (AL) were not initially affected, although plague apparently spread overland to these states, presumably from PE, by 1912 and 1914, respectively [6]. Extensive control efforts subsequently led to the virtual disappearance of plague from urban centers. However, by the 1930s, plague had transitioned to rural areas and become established in various enzootic foci, affecting native rodents and their fleas. Coinciding with this change, human cases decreased markedly, from an average of ~188 per year between 1899 and 1929, to ~20–100 per year up until the mid-1980s. Since then, only three human cases in the 1990s and one case in 2005 have been reported, although serological surveys suggest ongoing human plague activity in known foci [6].

Ongoing plague surveillance activity in Brazil led to the accumulation of 907 *Y*. *pestis* strains that were isolated between 1966 and 1997, and are maintained by the Serviço de Referência Nacional em Peste of the Instituto Aggeu Magalhães (SRP/IAM) in Recife, PE [6]. Assorted subtyping efforts on subsets of this collection have had varying levels of success at differentiating among strains. Early efforts involving phenotypic assays and plasmid analysis were unable to differentiate among strains [7, 8]. Pulsed-field gel electrophoresis (PFGE) was more successful, identifying 19 pulsotypes among 22 strains, but was unable to identify any strong geographic correlations [9]. Clustered regularly interspaced palindromic repeats (CRISPR) analysis identified a limited number of unique CRISPR genotypes among 128 strains from 5 plague foci and supported a single introduction to Brazil. However, most of the analyzed strains were still indistinguishable [10]. In contrast, an analysis of 20 *Y*. *pestis* strains from an epizootic event in Sítio Alagoinha in 1967 and 17 strains from an outbreak in Planalto da Borborema in 1986 using 12 variable-number tandem repeat (VNTR) loci provided 100% discrimination and identified three genetic groups, which revealed some association with the geographic/temporal origin of the strains [11]. Here, we expand upon these previous analyses using whole genome sequencing. We sequenced a total of 411 strains from six Brazilian foci that were isolated from 1966 to 1997 and examined phylogeographic patterns among these strains.

## Materials and methods

### DNAs

DNA was extracted from 411 *Y*. *pestis* strains belonging to the *Y*. *pestis* culture collection (FIOCRUZ–CYP) of the SRP/IAM [12] using the QIAGEN DNeasy Blood and Tissue Kit (QIAGEN, Inc., Germantown, MD). The strains were isolated from 1966 to 1997 from six plague foci in Brazil and include strains isolated from humans, rodents, and fleas (S1 Table). These strains were originally isolated from human clinical specimens (bubo fluid, blood), tissues (spleen, liver, blood) collected from rodents suspected of being infected with *Y*. *pestis*, and fleas obtained from humans or captured animal hosts using standard bacteriological methods [13, 14]. DNAs extracted from human derived strains were de-linked from the patients from whom they originated and analyzed anonymously.

### Whole genome sequencing

Whole genome sequencing was performed on all 411 Brazilian *Y*. *pestis* strains. Genomic DNA quality and quantity were evaluated by 0.7% agarose gel analysis. DNA samples (~1 μg/sample) were then fragmented using a SonicMan (Matrical, Inc., Spokane, WA) with the following parameters: 75 s pre chill, 16 cycles, 10 s sonication, 100% power, 75 s lid chill, 10 s plate chill, and 75 s post chill. The fragmented DNA was then size selected to target 600–650 bp by fragment separation using Agencourt AMPure XP beads (Beckman Coulter, Code A63882, Indianapolis, IN) and eluted into 42.5 μl of Elution Buffer. The KAPA Library Preparation Kit with SRPI Solution and the Standard PCR Library Amplification/Illumina series (KAPA Biosystems, Code KK8232, Wilmington, MA) were used for library preparation. Three separate library preparation reactions for each sample were carried out as follows: 1) End repair: 2.5 μl Enzyme, 5 μl Buffer, 1.6X AMPure XP, 43.5 μl Elution buffer, 2) A-Tailing: 1.5 μl Enzyme, 5 μl Buffer, 1.6X AMPure XP, 36.5 μl Elution buffer and 3) Quick Ligation: 2.5 μl Enzyme, 10 μl Buffer, 0.9X AMPure XP, 30 μl Elution buffer. The adapter ligation step used 1 μl of the 10 μM adapter oligo mix [15]. These reactions included the following modifications to the manufacturer’s protocol: 1) adapters and 8 bp index oligos based on Kozarewa and Turner (2011) [15] were purchased from IDT (Integrated DNA Technologies, San Diego, CA) and used in place of those supplied in the KAPA preparation kit to utilize a previously described dual indexing approach [16], 2) during the enzymatic steps, half the enzyme volume was used with the full volume of buffer as described in the KAPA Library Preparation protocol, and 3) The PCR was optimized to improve yield and genome coverage and included 2 μl of DNA, 2 μl of each 10 μM indexing primer, 25 μl of KAPA 2X HIFI PCR Master Mix (KAPA Biosystems), and 19 μl of molecular grade water, with the following PCR parameters: initial denaturation 2 min at 98°C, 8 cycles of 30 s at 98°C, 20 s at 65°C, 30 s at 72°C, and a final extension of 5 min at 72°C. The libraries were purified with a 0.9X AMPure XP bead cleanup and eluted into 50 μl of Elution Buffer. The final libraries were quantified using the KAPA ABI Prism Library Quantification Kit (KAPA Biosystems, Code KK4835) and pooled together at equimolar concentrations. The pool was quantified using the KAPA ABI Prism Library Quantification Kit, and the quality of the pool assessed with a Bioanalyzer DNA 1000 chip (Agilent Technologies, Code 5067–1504, Santa Clara, CA). Whole genome sequencing was then performed on an Illumina HiSeq 2000 (Illumina, Inc., San Diego, CA) using the 200-cycle TruSeq SBS Kit v3-HS (Illumina, Code FC-401-3001) with the standard Illumina procedure.

#### SNP calling and phylogenetics

**Fig 1:**
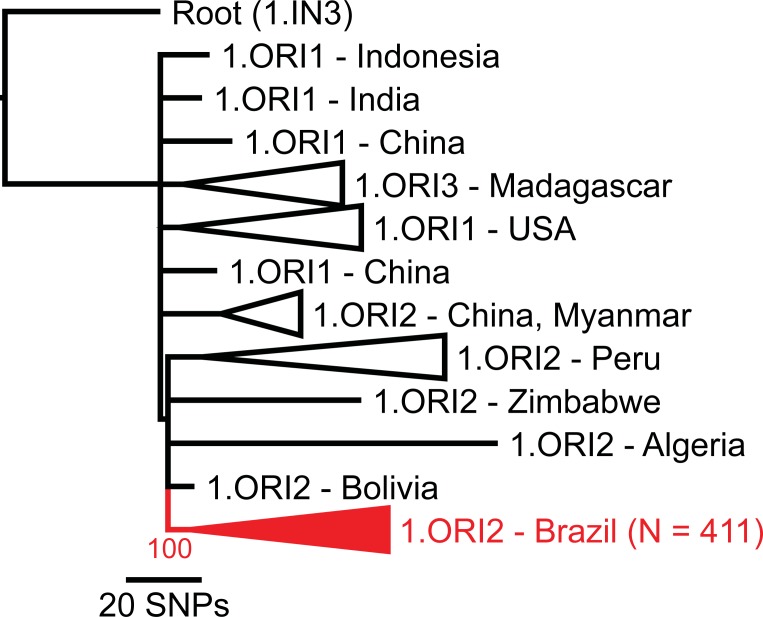
*Yersinia pestis* 1.ORI population phylogeny. Maximum parsimony phylogeny based on single nucleotide polymorphisms (SNPs) identified among 461 *Y*. *pestis* genomes belonging to the 1.ORI population, including 411 Brazilian *Y*. *pestis* strains (S1 Table, S2 Table). All 411 Brazilian *Y*. *pestis* genomes were assigned to a single monophyletic clade (red) with 100% bootstrap support. The phylogeny was rooted on strain D106004 (Genbank accession No. GCA 000022805.1), a member of the 1.IN3 *Y*. *pestis* population.

**Fig 2:**
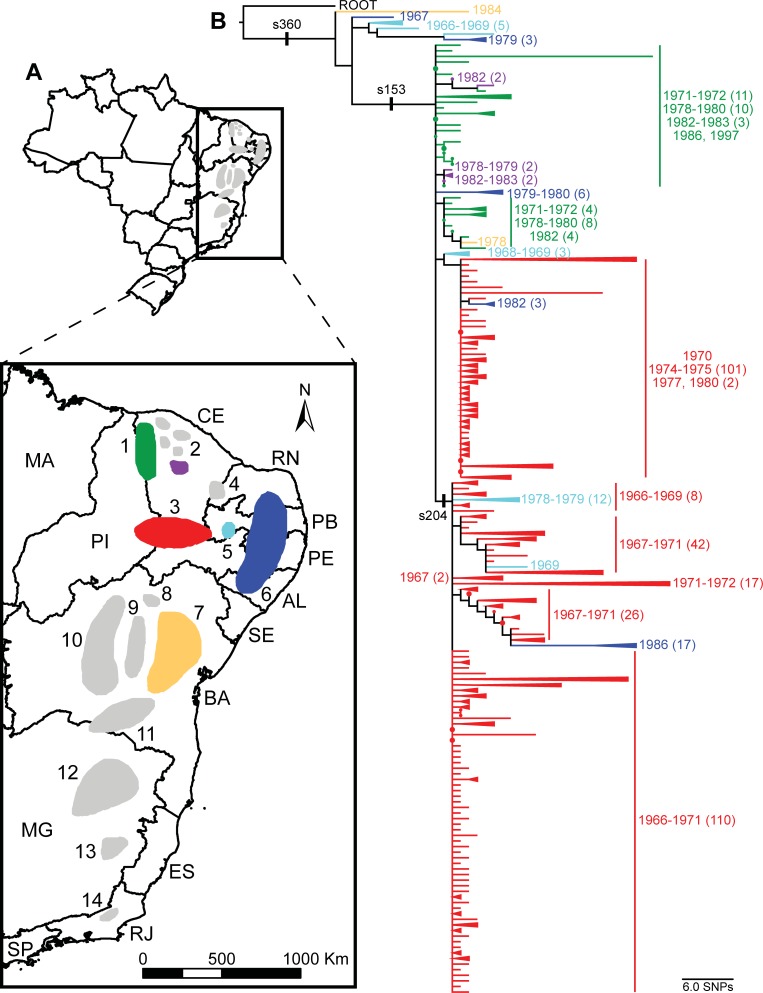
Phylogeography of *Yersinia pestis* in Brazil. (A) Map indicating recognized plague foci in Brazil. The six foci represented in this study are shown in color with unrepresented foci in gray. Foci are as follows: 1) Serra de Ibiapaba (green), 2) Serra das Matas, de Baturité (purple), de Pedra Branca, do Machado, and de Uruburetama, (these five smaller foci are sometimes grouped together as a single focus) 3) Chapada do Araripe (red), 4) Chapada do Apodi, 5) Serra de Triunfo (light blue), 6) Chapada da Borborema (dark blue), 7) Planalto Oriental da Bahia (orange), 8) Serra do Formoso, 9) Piemonte da Diamantina, 10) Chapada Diamantina, 11) Planalto da Conquista, 12) Vale do Jequitinhonha, 13) Vale do Rio Doce, and 14) Serra dos Órgãos [6]. The Brazilian state abbreviations are also indicated. (B) Maximum parsimony phylogeny based on single nucleotide polymorphisms (SNPs) identified among 411 *Y*. *pestis* genomes from Brazil and rooted on *Y*. *pestis* strain CO92. Clades containing strains isolated from the same focus and in the same or close years were collapsed to simplify the tree. Branches and clades are colored to indicate the focus of origin. The date of isolation (presented as a year or range of years, as appropriate) is indicated for groups of strains with the same focus of origin. Numbers in parentheses following each listed year or range indicate the number of strains isolated in that year or range when the number of strains is >1. The position of three 1.ORI2 SNPs from Morelli et al. [2] are also indicated on the phylogeny.

Raw reads were aligned against the completed genome of the CO92 strain of *Y*. *pestis* [17] with BWA-MEM [18] and all single nucleotide polymorphisms (SNPs) were called with the UnifiedGenotyper method in GATK [19, 20]; these methods were wrapped by the NASP pipeline [21]. SNPs were filtered from downstream analyses if they fell within duplicated regions of the reference genome based on a NUCmer [22] self-alignment, or if the SNPs had a depth of coverage <3X or an allele proportion <90%. Phangorn [23] was used to generate a maximum parsimony phylogeny, including 100 bootstrap replicates, from the concatenated SNP alignments of the Brazilian *Y*. *pestis* strain genomes, all publicly available 1.ORI *Y*. *pestis* whole genome sequences, and a 1.IN3 *Y*. *pestis* genome as a root (Fig 1). Confirmation of the placement of the Brazilian *Y*. *pestis* strains within the 1.ORI2 subpopulation suggested in Fig 1 was determined by querying relevant 1.ORI2 SNPs from Morelli et al. [2] (s36, s37, s38, s40, s47, s121, s151, s153, s162, s163, s164, s168, s169, s170, s172, s176, s179, s182, s184, s186, s189, s191, s194, s200, s202, s203, s204, s205, s207, s225, s240, s255, s281, s311, s360, s649, s841, and s1230) against the Brazilian *Y*. *pestis* whole genome sequence data. Phangorn was also used to generate a second maximum parsimony phylogeny of just the Brazilian *Y*. *pestis* strain genomes with *Y*. *pestis* strain CO92 as a root (Fig 2B). This second maximum parsimony phylogeny was color coded to reflect the Brazilian plague foci of origin described by Tavares et al. [6] and elucidate the phylogeography of the Brazilian *Y*. *pestis* strains (Fig 2).

#### Genome assembly and comparative genomics

Genomes were assembled with SPAdes v3.11.1 [24]. To identify differential conserved regions, a large-scale blast score ratio (LS-BSR) analysis [25] was performed. Coding region sequences (CDSs) were identified with Prodigal v2.60 [26] and clustered with USEARCH v10.0 [27]. Each unique peptide, based on a clustering at 90% amino acid identity, was aligned against Prodigal predicted coding regions with Diamond v0.9.10.111 [28]. Sixty-three peptides that showed variable distribution across the phylogeny, as demonstrated by the BSR value [29], along with the phylogeny, were visualized with the interactive tree of life [30] and were annotated by the best BLASTP [31] alignment against the GenBank [32] nr database.

## Results and discussion

The plague foci in Brazil are the result of a single introduction of *Y*. *pestis* during the third pandemic that became successfully established in Brazil. All 411 Brazilian *Y*. *pestis* strains were assigned to a single, strongly supported, monophyletic clade within the 1.ORI population (Fig 1), indicating that there was only a single *Y*. *pestis* introduction to Brazil that led to the successful establishment of endemic foci. Importantly, this monophyletic clade only included strains from Brazil (Fig 1). Available strains of *Y*. *pestis* from other South American countries (Peru and Bolivia) were assigned to other clades within the 1.ORI population (Fig 1), suggesting that *Y*. *pestis* in these other countries is due to separate introduction events during the third pandemic. These results are consistent with a previous report by Morelli et al. [2], which placed two *Y*. *pestis* isolates from Brazil into the 1.ORI2.f node of a worldwide phylogeny. Querying the 1.ORI2 SNPs from Morelli et al. [2] placed the Brazilian *Y*. *pestis* genome sequences described here into nodes 1.ORI2.a (N = 10), a new node intermediate between SNP nodes 1.ORI2.a and 1.ORI2.f (1.ORI2.f1, N = 166), and node 1.ORI2.f (renamed here as 1.ORI2.f2, N = 235) (S1 Table). These results suggest that the introduction of *Y*. *pestis* to Brazil represents one monophyletic branch in the still unresolved polytomy of the 1.ORI2 subpopulation. Analysis of additional 1.ORI2 strains may provide additional insight into the spread of this 1.ORI subpopulation.

Following its introduction to Brazil, *Y*. *pestis* underwent a radial expansion, spreading rapidly to the interior and becoming ecologically established in several foci. The geographic introduction point for *Y*. *pestis* to Brazil remains unknown, but it may have been any of several port cities that experienced outbreaks in the early 1900s, including the commonly reported entry point of Santos, SP [6]. Regardless of the exact entry point, the lack of population structure indicated by the WGS data (Fig 2) suggests *Y*. *pestis* rapidly dispersed from the coast to the interior of Brazil, followed by localized differentiation. There are very few shared characters (i.e., SNPs) among the 411 Brazilian *Y*. *pestis* strains. Rather, most SNPs are found only in single strains (Fig 2). This low level of population structure is consistent with a radial expansion, which is a common feature in the evolutionary history of *Y*. *pestis* [1].

The lack of population structure evident in the WGS data extended to the phylogeographic analysis. Overall, there were no strong spatial or temporal patterns among the 411 Brazilian *Y*. *pestis* genomes, although there were some general trends. First, in general, strains from the same focus tended to cluster together. There were some exceptions to this, most notably for strains from the Serra de Triunfo and Chapada da Borborema foci (Fig 2, light blue and dark blue foci). Strains from these foci were found on multiple branches in the phylogeny, which may be indicative of *Y*. *pestis* movement among foci. Second, strains isolated from foci closest to the coastal regions tended to fall in more basal positions in the WGS tree, with strains isolated from the more interior foci falling in more derived positions. Although there are some exceptions to these trends, these patterns are consistent with a rapid spread from the coastal regions to the interior, followed by ecological establishment in these foci.

**Fig 3:**
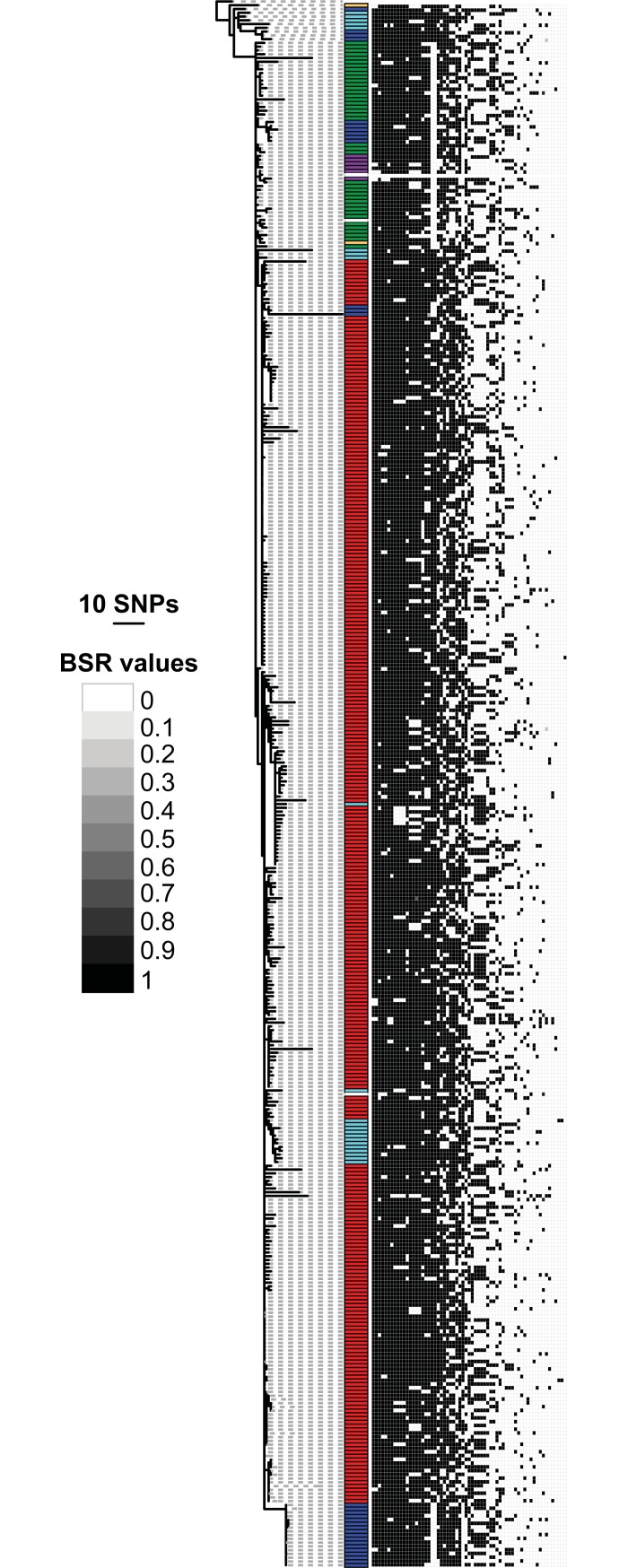
CDS variability among Brazilian strains. The expanded maximum parsimony phylogeny from Fig 2 is presented with a heatmap generated by blast score ratio (BSR) values and visualized with the Interactive Tree of Life (iTOL). The BSR values range from 0 (no significant alignment) to 1 (identical alignment) and demonstrate the variability in gene content among the Brazilian strain genomes. Colored bars indicate the focus of origin for each strain as in Fig 2.

The LS-BSR analysis was consistent with the SNP analysis. Both analyses revealed a moderate level of genetic diversity among the 411 Brazilian *Y*. *pestis* strains, with 822 SNPs and 63 variably distributed CDSs identified (Figs 2 and 3, S3 Table). Similar to the SNP analysis, there were few patterns among the identified CDSs (Fig 3), reflecting the limited population structure among these Brazilian *Y*. *pestis* strains. The LS-BSR analysis indicated mostly variation in the presence or absence of the identified CDSs rather than sequence differences (Fig 3). Such gene loss is not uncommon in *Y*. *pestis*, which is a recently emerged clone of *Y*. *pseudotuberculosis* that exhibits genomic reduction consistent with its transition to an obligate vector-borne pathogen [33]. Indeed, few to none of the CDSs identified in the LS-BSR analysis appear likely to be under selection (S3 Table). This suggests that these CDS differences are simply the result of neutral variation associated with the localized differentiation of *Y*. *pestis* following its radial expansion in Brazil.

Unfortunately, the lack of strains from many of the Brazilian plague foci prevented a comprehensive analysis of *Y*. *pestis* phylogeography in Brazil. Starting in the 1930s, most human plague cases in Brazil occurred in extremely remote rural areas, and the last recorded urban cases occurred in the first half of the 1960s [6]. In contrast, routine *Y*. *pestis* culture collection did not start until the mid-1960s [6, 12]. As such, the available strain diversity is limited, with the majority of strains originating from the most active northeastern plague foci, including the six foci represented here. Despite the declining number of human cases in Brazil, residual human plague activity has continued to be detected in focal areas using serology [6]. In addition, ecological niche modeling [34] is consistent with the identified Brazilian plague foci [6], suggesting that these regions contain suitable ecological conditions for the persistence of *Y*. *pestis*. Additional whole genome sequences from more modern strains might reveal additional phylogeographic patterns.

Overall, the patterns observed in Brazil are similar to other locations affected during the 3rd pandemic. Globally, the *Y*. *pestis* phylogeny is characterized by several polytomies, reflecting a series of nested radial expansions in the history of this species. These include a major polytomy at the base of the 1.ORI population responsible for the third pandemic and other radial expansions within that population [1]. Among these are radial expansions associated with the establishment of plague foci in North America and Madagascar [1, 2], and now Brazil. *Y*. *pestis* from each of these locations exhibits patterns indicative of an initial rapid radial expansion following a single successful introduction. This rapid spread was then followed, in turn, by ecological establishment in rodent reservoirs and long term enzootic persistence [2, 35]. Such rapid epizootic spread followed by enzootic maintenance is a hallmark of *Y*. *pestis* as a species [1, 2, 4, 35, 36].

## Supporting information

S1 Table Brazilian *Yersinia pestis* strains. (XLSX)

S2 Table Reference genomes used to generate Fig 1. (XLSX)

S3 Table CDS identified in LS-BSR analysis presented in Fig 3. (XLSX)
